# Effect of Dielectric Distributed Bragg Reflector on Electrical and Optical Properties of GaN-Based Flip-Chip Light-Emitting Diodes

**DOI:** 10.3390/mi9120650

**Published:** 2018-12-08

**Authors:** Shengjun Zhou, Haohao Xu, Mengling Liu, Xingtong Liu, Jie Zhao, Ning Li, Sheng Liu

**Affiliations:** 1 Key Laboratory of Hydraulic Machinery Transients, Ministry of Education, Wuhan University, Wuhan 430072, China; 2017202080015@whu.edu.cn (H.X.); lml0305@whu.edu.cn (M.L.); 2016202080010@whu.edu.cn (X.L.); 2017202080016@whu.edu.cn (J.Z.); 2017282080133@whu.edu.cn (N.L.); 2 Center for Photonic and Semiconductor, School of Power and Mechanical Engineering, Wuhan University, Wuhan 430072, China; 3 Research Center of Electronic Manufacturing and Packaging Integration, Institute of Technological Sciences, Wuhan University, Wuhan 430072, China; 4 State Key Laboratory of Applied Optics, Changchun Institute of Optics, Fine Mechanics and Physics, Chinese Academy of Sciences, Changchun 130033, China

**Keywords:** flip-chip light-emitting diodes, distributed Bragg reflector, light output power, external quantum efficiency

## Abstract

We demonstrated two types of GaN-based flip-chip light-emitting diodes (FCLEDs) with distributed Bragg reflector (DBR) and without DBR to investigate the effect of dielectric TiO_2_/SiO_2_ DBR on optical and electrical characteristics of FCLEDs. The reflector consisting of two single TiO_2_/SiO_2_ DBR stacks optimized for different central wavelengths demonstrates a broader reflectance bandwidth and a less dependence of reflectance on the incident angle of light. As a result, the light output power (LOP) of FCLED with DBR shows 25.3% higher than that of FCLED without DBR at 150 mA. However, due to the better heat dissipation of FCLED without DBR, it was found that the light output saturation current shifted from 268 A/cm^2^ for FCLED with DBR to 296 A/cm^2^ for FCLED without DBR. We found that the use of via-hole-based *n*-type contacts can spread injection current uniformly over the entire active emitting region. Our study paves the way for application of DBR and via-hole-based *n*-type contact in high-efficiency FCLEDs.

## 1. Introduction

The wide bandgap GaN and related materials have been extensively studied and implemented for optoelectronic devices that emit light in the spectrum between ultraviolet and visible light [1,2,3,4,5,6,7]. GaN-based light-emitting diodes (LEDs) have been extensively adopted in a number of applications such as high-resolution micro-displays, automotive lighting, optogenetics, visible light communication (VLC), and solid-state lighting. [8,9,10,11,12,13,14]. The progress in the LED development has been attributed to significant improvement in device efficiency [15,16]. To further enhance the performance of LEDs, there is a great need to improve both internal quantum efficiency (IQE) and light extraction efficiency (LEE). The improvement of IQE has played a key role in LED development. Specifically, the IQE of the InGaN LEDs have been improved by using the large overlap quantum well concept or the new active material concept [17,18,19,20]. Generally, LEDs should be driven at a high current density to obtain higher light output power (LOP), which also inevitably generates a large portion of heat [21]. Increasing the operating current density of LEDs is also an effective method to decrease the carrier lifetime and increase the modulation bandwidth of VLC [22,23]. However, GaN-based top-emitting LEDs grown on sapphire substrate suffer from inferior heat dissipation performance due to the poor thermal conductivity of sapphire substrate [24,25]. Additionally, the LEE of top-emitting LEDs was limited by the absorption of light by opaque metal electrodes and total internal reflection (TIR) of the generated light at the GaN (n = 2.45)/air (n = 1) interface resulting from their very different refractive indices [26,27,28,29]. The vertical structure LEDs fabricated on a substrate with high thermal conductivity (such as Cu) can overcome the thermal issues. However, wafer bonding and laser lift-off techniques, which are critical fabrication processes for vertical structure LEDs, suffer from low-yield and high-cost [30,31,32,33]. The flip-chip technology was brought up to overcome these problems. The LEE of flip-chip LEDs (FCLEDs) was relatively higher compared with the top-emitting LED because of lower refraction index contrast between the sapphire (n = 1.77) and air (n = 1) [34,35]. The FCLEDs can also avoid light absorption by the opaque metal electrodes because light is extracted through sapphire substrate [36,37]. Furthermore, FCLEDs are commonly bonded to a high thermal conductivity submount such as silicon, resulting in a superior heat dissipation capability and a higher light output saturation current density. Accordingly, the FCLEDs can effectively improve modulation bandwidths of VLC since the FCLEDs can be operated at a higher injection current density as compared to top-emitting LEDs.

Photons generated from InGaN/GaN multiple quantum wells (MQWs) active region of FCLEDs emit in any direction. As a result, a large portion of photons emitted from the active region will be lost, particularly for those photons emitted downward. Thus, depositing a reflector onto *p*-GaN in order to reflect photons emitted downward can significantly enhance the LEE of the FCLEDs [38]. It has been reported that various reflectors, such as metallic mirrors and dielectric distributed Bragg reflectors (DBRs), have been used to enhance the LEE of FCLEDs [39,40,41,42]. In addition, highly reflective DBRs are also important for realizing high performance vertical-cavity surface emitting laser [43,44]. The metallic mirrors have high reflectivity in the visible wavelength range. However, metallic mirrors including Al and Ag suffer from inferior ohmic contact behavior and poor adhesion to the *p*-GaN layer. As an alternative to metallic reflector, the dielectric DBR has many advantages over a metallic reflector, such as low optical loss, high reflectance, and high mechanical robustness [45,46].

In this study, indium-tin oxide (ITO) transparent conductive layer combined with dielectric DBR is used as reflective *p*-type ohmic contact for FCLEDs, which leads to a significant reduction in absorption of light by opaque metal electrodes. We investigated the effect of dielectric TiO_2_/SiO_2_ DBR on the electrical and optical properties of FCLEDs. The dielectric DBR is composed of 14 alternating nanometer-thick layers of silicon dioxide (SiO_2_) and titanium dioxide (TiO_2_), which demonstrates high reflectance over the wavelength range from 400 nm to 650 nm at normal incidence. As a result, the light output power of FCLED with DBR was 25.3% higher than that of FCLED without DBR at 150 mA. In addition, via-hole-based *n*-type contacts were used to spread injection current uniformly over the entire active emitting region of FCLEDs. 

## 2. Materials and Methods 

GaN epitaxial layers were grown on c-plane (0001) patterned sapphire substrate (PSS) by metal organic chemical vapor deposition (MOCVD). The GaN-based LED structure consists of a 20-nm-thick low-temperature GaN nucleation layer, a 3.0-μm-thick undoped GaN buffer layer, a 2.5-μm-thick Si-doped *n*-GaN layer, a 16-pair In_0.02_Ga_0.98_N (2.1 nm)/GaN (2.3 nm) superlattice, a 12-pair In_0.16_Ga_0.84_N (3 nm)/GaN (12 nm) multiple quantum wells (MQWs), a 20-nm-thick low-temperature *p*-GaN layer, a 45-nm-thick *p*-Al_0.15_Ga_0.85_N electron blocking layer, and a 120-nm-thick Mg-doped *p*-GaN layer. The LED wafer was subsequently annealed at 750 °C at N_2_ atmosphere to activate Mg acceptor in the *p*-GaN. The peak wavelength of FCLEDs is 465 nm.

**Figure 1:**
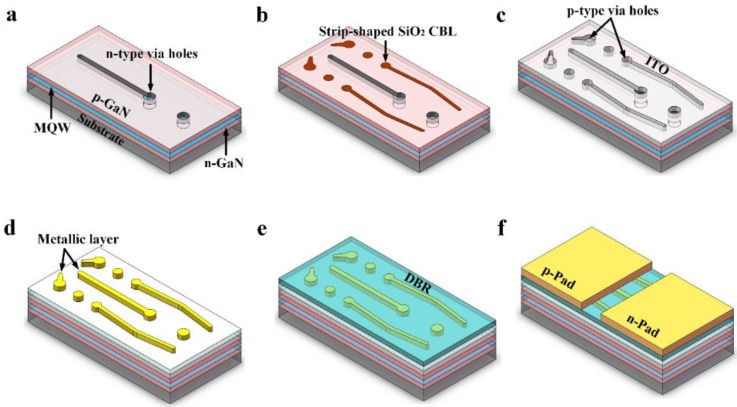
Schematic illustration of the fabrication process for a FCLED with DBR.

**Figure 2:**
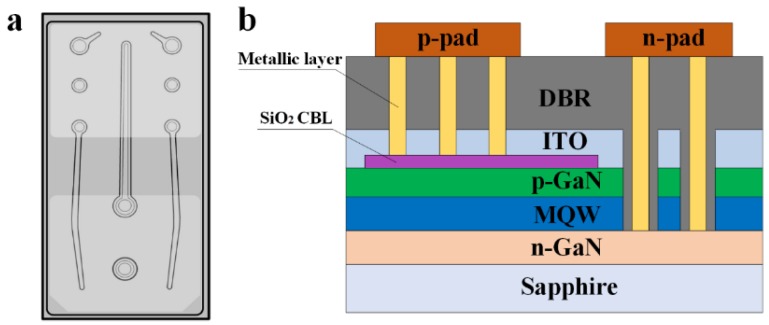
Schematic illustration of a FCLED with DBR: (**a**) Top-view image. (**b**) Cross-section image.

Figure 1 shows a schematic illustration of the fabrication processes for FCLED with DBR. The detailed fabrication processes were shown as follows: a. First, an inductively coupled plasma (ICP) etching based on BCl_3_/Cl_2_/Ar gas chemistry was used to form *n*-type via holes by etching a portion of the *p*-GaN and the InGaN/GaN MQWs to expose the *n*-GaN layer. b. A 200-nm-thick SiO_2_ was then deposited on the *p*-GaN layer by plasma enhanced chemical vapor deposition (PECVD), followed by optical photolithography and buffered oxide etch (BOE) wet etching process to form strip-shaped SiO_2_ current blocking layer (CBL). c. A 115-nm-thick ITO was deposited on the *p*-GaN as a *p*-type ohmic contact using electronic beam evaporator, followed by thermal annealing at 550 °C under N_2_ ambient. d. Next, Cr/Al/Ti/Pt/Au (20 nm/100 nm/50 nm/50 nm/1 μm) metal was deposited on the ITO and *n*-GaN layers to form the *p*- and *n*-electrodes. e. DBR consisting of 14 alternating pairs of TiO_2_/SiO_2_ was deposited on the ITO by ion beam sputtering. f. Cr/Al/Ti/Pt/Ti/Pt/Au (20 nm/100 nm/50 nm/50 nm/50 nm/50 nm/1 μm) layers were then evaporated into *p*-type via holes and *n*-type via holes as *p*- and *n*-pads. Finally, the LED wafers were thinned down to be about 150 μm and diced into chips with a dimension of 380 μm × 760 μm. The schematic illustration of the FC-LED with DBR is shown in Figure 2. FCLED without DBR was also fabricated for comparison. For device characterization, current–voltage (I–V) characteristics were measured by using a semiconductor parameter analyzer (Keysight B2901A). The light output power (LOP)–current (L-I) characteristics of LED were determined using a calibrated integrating sphere. The light emission images of LED were obtained using a calibrated charge-coupled device (CCD) camera mounted on a microscope. 

## 3. Results and Discussion

**Figure 3:**
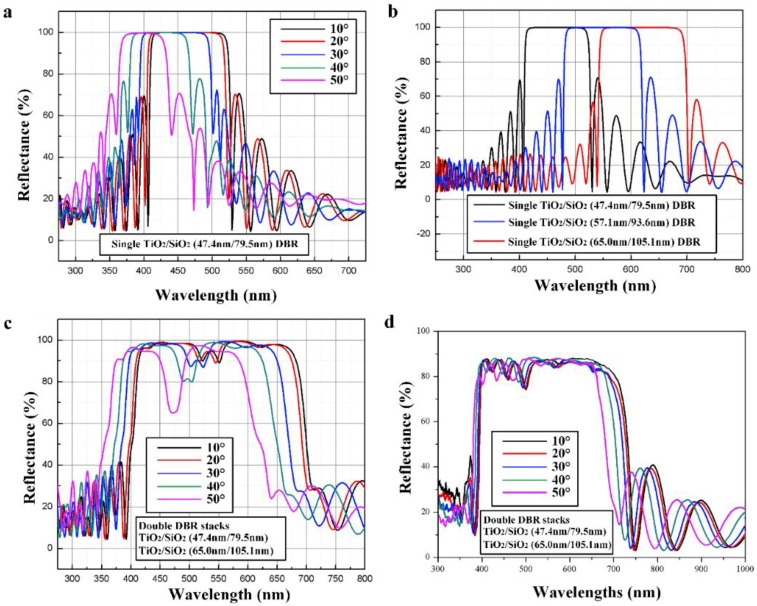
(**a**) Reflectance spectra of the single DBR stack as a function of incident angles of light. (**b**) Normal-incident reflectance spectra of the single TiO_2_/SiO_2_ DBR stack optimized for a different central wavelength. (**c**) Reflectance spectra of the double DBR stacks as a function of incident angles of light. (**d**) Measured reflectance spectra of double DBR stacks.

We used the commercial software, TFCalc, to model the design of a conventional single DBR stack consisting of 14 pairs of TiO_2_/SiO_2_ dielectric layers optimized for central wavelength at 465 nm. In the simulation, the refractive indices of the SiO_2_/TiO_2_ were fixed at 1.45/2.55, and the thicknesses of the SiO_2_/TiO_2_ were fixed at 47.4 nm/79.5 nm. Figure 3a shows reflectance spectra of the 14 pairs of single TiO_2_ (47.4 nm) /SiO_2_ (79.5 nm) DBR stack as a function of incident angles of light. It was clearly observed that the reflective bandwidth was narrowed and blueshifted toward the short wavelength when the incident angle of light was increased. Figure 3b shows normal-incident reflectance spectra of the single TiO_2_/SiO_2_ DBR stack optimized for central wavelength of 465 nm, 545 nm, and 620 nm. The thickness of TiO_2_/SiO_2_ dielectric layers was 47.4 nm/79.5 nm, 64.29 nm/92.73 nm, and 65.0 nm/105.1 nm. As the thickness of the TiO_2_/SiO_2_ dielectric layers was increased, the reflective bandwidth of the single DBR stack was redshifted. The redshift toward the long wavelength for the single DBR stack with increasing thickness of TiO_2_/SiO_2_ dielectric layers can counteract the blueshift toward the short wavelength when the incident angle of light increased from the surface normal toward the grazing angle to the DBR stack [47]. To obtain a larger bandwidth of reflectance band and less dependence on incident angles of light, we combined two single DBR stacks into double DBR stacks. Each single TiO_2_/SiO_2_ DBR stack was optimized for a different central wavelength. The first DBR stack is composed of seven pairs of TiO_2_/SiO_2_ (47.4 nm/79.5 nm) dielectric layers optimized for a central wavelength at 465 nm; the second DBR stack consists of another seven pairs of TiO_2_/SiO_2_ (65.0 nm/105.1 nm) dielectric layers optimized for a central wavelength at 620 nm. Figure 3c shows the reflectance spectra of the double DBR stacks as a function of incident angles of light. We find that as the incident light deviates from normal incidence, the blueshift of the double DBR stack is not obvious. This phenomenon indicates that the double DBR stacks exhibit less angular dependence as compared to the single DBR stack. Figure 3d shows the measure reflectance spectra of double DBR stacks. The measured reflectance bandwidth of double DBR stacks was in good agreement with the result of numerical simulation. 

**Figure 4:**
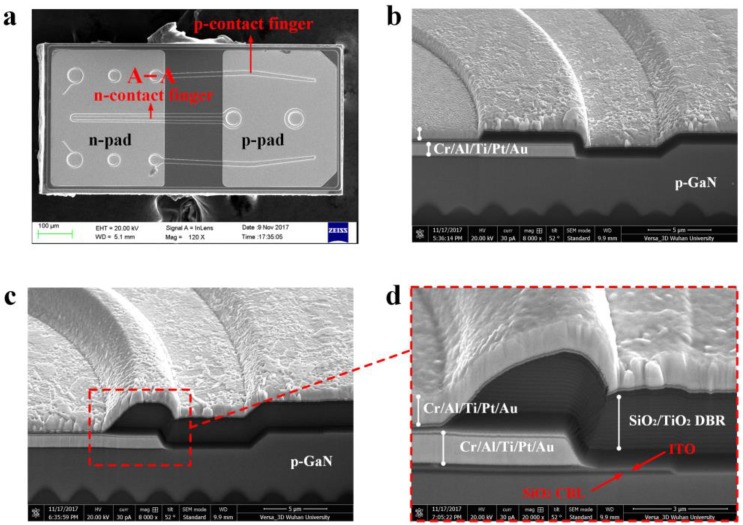
(**a**) Top-view SEM image of the fabricated FCLED with DBR. (**b**) Cross-sectional SEM image of the FCLED without DBR. (**c**) Cross-sectional SEM image of the FCLED with DBR. (**d**) Magnified Cross-sectional SEM image of the FCLED with DBR.

Figure 4a shows the top-view SEM images of the FCLED with DBR. The electrode pattern of FCLED without DBR is the same as that of FCLED with DBR. For the FCLEDs with and without DBR, two *p*-contact fingers are finely distributed on both sides of the *n*-contact finger, which can improve the uniformity of current spreading over the active region by reducing lateral current spreading distance between the *p*-type contact and the *n*-type contact. Figure 4b,c show the cross-sectional SEM images of the FCLED with and without DBR, which were obtained by focused ion beam milling along the A-A direction, as shown in Figure 4a. Figure 4d shows magnified cross-sectional SEM image of the FCLED with DBR. In Figure 4d, contact to ITO was obtained by the formation of *p*-type via holes through dielectric TiO_2_/SiO_2_ DBR. The sheet resistance of 115-nm-thick ITO film (36 Ω/sq) is much larger than that of the as-grown *n*-GaN layer (18 Ω/sq), resulting in severe current crowding at the edge of the *p*-contact electrode. Therefore, a strip-shaped SiO_2_ CBL underneath the *p*-electrode was implemented to further improve current spreading of FCLEDs. 

**Figure 5:**
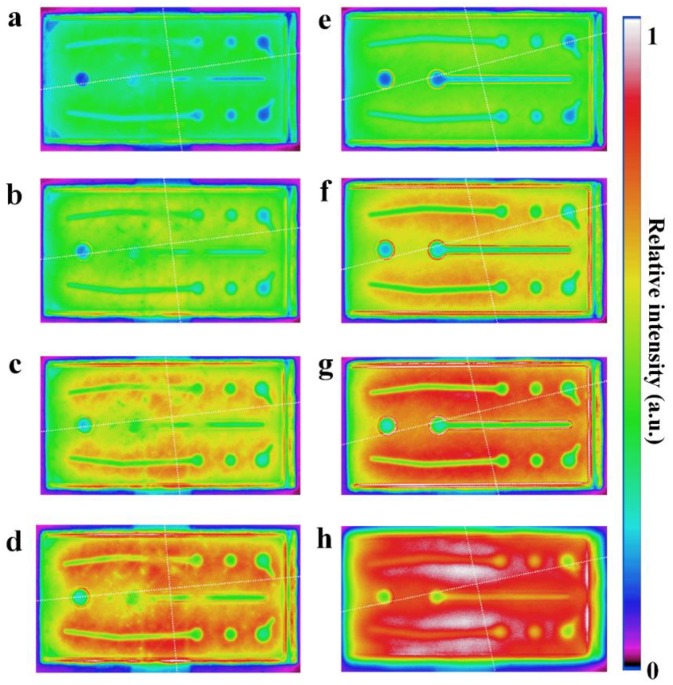
(**a**–**d**) Light emission intensity distribution images of FCLED without DBR at 100 mA, 150 mA, 200 mA and 250 mA. (**e**–**h**) Light emission intensity distribution images of FCLED with DBR at 100 mA, 150 mA, 200 mA and 250 mA.

Figure 5 shows spatial distribution of light emission intensity images of FCLEDS with and without DBR measured by a calibrated CCD camera. Figure 5a–d show the light emission intensity distribution images of FCLED without DBR at 100 mA, 150 mA, 200 mA and 250 mA. Figure 5e–h show the light emission intensity distribution images of FCLED with DBR at 100 mA, 150 mA, 200 mA and 250 mA. As a higher current density can cause a stronger light emission intensity, the spatial distribution of light emission intensity is closely related to the distribution of current density. We observed that the light emission intensity of FCLEDs increased with the increase of injection current density. The current crowding occurring in both FCLEDs is not obvious at 100 mA, as shown in Figure 5. As the injection current is further increased, the current congregated near the *p*-electrode of FCLEDs, leading to non-uniform light emission intensity in both FCLEDs. It was clearly indicated that the FCLED with DBR exhibited a stronger light emission intensity due to the use of dielectric TiO_2_/SiO_2_ DBR having high reflectance. 

**Figure 6:**
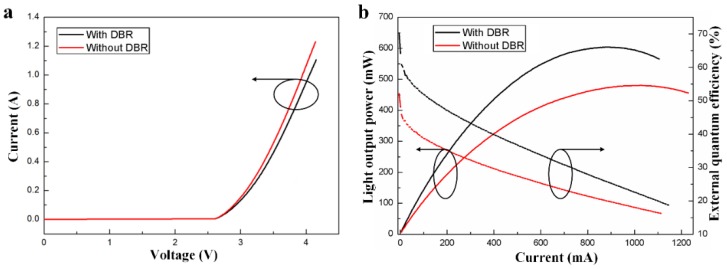
(**a**) Current versus voltage of FCLEDs with and without DBR. (**b**) Light output power versus current and EQE versus current characteristics of FCLEDs with DBR and without DBR.

The current versus voltage for the FCLEDs with and without DBR is shown in Figure 6a. At 150 mA, the forward voltages of the FCLEDs with and without DBR were 3.11 V and 3.03 V, respectively. The LOP versus current and external quantum efficiency (EQE) versus current characteristics of the FCLEDs with and without DBR were shown in Figure 6b. At 150 mA, the LOPs of the FCLEDs with DBR and without DBR were 204.6 mW and 152.8 mW, respectively. The LOP of the FCLED with DBR was 25.3% higher than that of the FCLED without DBR at 150 mA. This result can be attributed to the use of DBR having high reflectance in blue light wavelength region. At 150 mA, the corresponding EQEs of the FCLEDs with DBR and without DBR were 49.5% and 39.0%, respectively. The EQE of the FCLED with DBR was 21.2% higher than that of the FCLED without DBR. The light output saturation currents of the FCLED with and without DBR were 875 mA and 1025 mA, respectively. As the dielectric TiO_2_/SiO_2_ DBR has low thermal conductivity, the FCLED without DBR exhibited higher light output saturation current compared to the FCLED with DBR, owing to a better heat dissipation performance in FCLED without DBR. 

## 4. Conclusions

In summary, we have compared the optical and electrical characteristics of FCLEDs with and without DBR. To reduce angular dependence of single DBR stack and increase reflectance bandwidth, we combined two single TiO_2_/SiO_2_ DBR stack into double DBR stacks. The double DBR stacks exhibited a high reflectance of 97.8% at 465 nm. Additionally, via-hole-based *n*-type contacts were used to improve current spreading of FCLEDs. As a result, the LOP of FCLED with DBR was 25.3% higher than that of the FCLED without DBR at 150 mA. However, owing to low thermal conductivity of dielectric TiO_2_/SiO_2_ DBR, FCLEDs with DBR exhibited lower light output saturation current as compared to FCLEDs without DBR.
